# Fructose in obesity and cognitive decline: is it the fructose or the excess energy?

**DOI:** 10.1186/1475-2891-13-27

**Published:** 2014-03-25

**Authors:** Laura Chiavaroli, Vanessa Ha, Russell J de Souza, Cyril WC Kendall, John L Sievenpiper

**Affiliations:** 1 Toronto 3D Knowledge Synthesis and Clinical Trials Unit, Clinical Nutrition and Risk Factor Modification Centre, St. Michael’s Hospital, Toronto, ON, Canada; 2 Department of Nutritional Sciences, Faculty of Medicine, University of Toronto, Toronto, ON, Canada; 3 Department of Clinical Epidemiology and Biostatistics, Faculty of Health Sciences, McMaster University, Hamilton, ON, Canada; 4 College of Pharmacy and Nutrition, University of Saskatchewan, Saskatoon, SK, Canada; 5 Department of Pathology and Molecular Medicine, Faculty of Health Sciences, McMaster University, Hamilton, ON, Canada; 6 Li Ka Shing Knowledge Institute, St. Michael's Hospital, Toronto, ON, Canada

**Keywords:** Dietary fructose, Cognitive decline, Obesity, Energy

## Abstract

We read with interest the review by Lakhan and Kirchgessner, proposing that high fructose intake promotes obesity, metabolic syndrome, diabetes, and cognitive decline. Their focus on the role of fructose seems premature due to confounding from energy and the heavy reliance on low quality evidence from animal models. There is a lack of high quality evidence directly assessing the role of fructose in cognitive decline. Although one cannot exclude the possibility of a link, it remains an unconfirmed hypothesis.

## Letters to the Editor

Dear Editor,

We read with interest the review by Lakhan and Kirchgessner [1], proposing that high fructose intake promotes obesity and its downstrean complications including metabolic syndrome, diabetes, and cognitive decline. We feel this focus on the role of fructose is premature, as the authors did not take into consideration important confounding from energy and relied heavily on weak evidence from animal models.

Any discussion of the role of fructose in obesity and its metabolic complications must take into consideration energy intake and balance. We have published several systematic reviews and meta-analyses of controlled feeding trials in humans of the effect of fructose on cardiometabolic risk factors, demonstrating that excess calories may be the dominant factor in mediating the effects attributed to fructose. We have found that when dietary fructose is consumed in isocaloric substitution for other carbohydrate sources (that is, where the fructose arm is matched for energy to the carbohydrate comparator), there are no deleterious effects with advantages seen for glycemic control and blood pressure [2-8]. An overall lack of harm is true even where fructose provides excess calories (positive energy balance), as long as the carbohdyate comparator is matched for the excess calories. Fructose, however, does show adverse signals for fasting lipids in some high dose subgroup analyses [8-10] and for most endpoints when it provides excess calories in imbalanced hypercaloric comparisons, in which fructose supplements control diets with excess calories compared to the same diets alone without the excess calories [2-7]. In the absence of consistent adverse effects in isocaloric comparisons, the consistent adverse effects seen in hypercaloric comparisons suggests that excess calories are the culprit. This view is supported by other high quality systematic reviews and meta-analyses of the effect of other fructose-containing sugars [11,12]. It is also supported by the secondary sources the authors themselves cited. Energy balance, therefore, remains an important confounder in assessing the role of fructose in obesity and its metabolic complications including cognitive decline.

One must be careful in placing too much weight on the biological plausibility provided by animal models in the fructose debate. The ability to translate these models to human physiology is limited. The majority of animal studies feed fructose at levels (typically ~60% of total energy) many fold higher than population levels of intake [13]. *De novo* lipogenesis, the main pathway through which fructose is thought to have its deleterious effects, also appears to be very different in animals. *De novo* lipogenesis from fructose accounts for 60-70% of fatty acids in rodents, while its contribution is quantitatively insignificant [13]. Two carefully conducted reviews of isotopic tracer studies showed that glucose (~50%), lactate (~25%), and glycogenesis (>15%) synthesis remain the major pathways of hepatic fructose disposal in humans, while *de novo* lipogenesis contributes <1% of fatty acids [14,15]. Although fructose, more than other sources of carbohydrate, may stimulate *de novo* lipogenesis, its ability to do so in a meaningful way may only be seen in hypercaloric comparisons, in which fructose supplements diets with excess calories at extreme doses.

Overall, we feel that the conclusions of this review should be viewed with the appropriate caution. Where we do have higher quality evidence of the effect of fructose on cardiometabolic risk factors associated with cognitive decline, there is significant evidence of confounding from energy. There is otherwise a lack of high quality evidence directly assessing the role of fructose in cognitive decline. Although one cannot exclude the possibility of a link, it remains an unconfirmed hypothesis.

## Competing interests

LC has received research support from the Canadian Institutes of Health Research (CIHR) and the Agricultural Bioproducts Innovation Program (ABIP) through the Pulse Research Network (PURENet) and the Saskatchewan Pulse Growers. She is also a clinical research coordinator at Glycemic Index Laboratories, Toronto, Ontario, Canada. VH has received a Province of Ontario Graduate Scholarship and research support from the CIHR and World Health Organization (WHO) for work on a systematic review and meta-analysis commissioned by the WHO of the relation of saturated fatty acids with health outcomes. She also received a travel award to attend the "Journey Through Science Day" hosted by PepsiCo and the New York Academy of Sciences (NYAS). RJdS is funded by a CIHR Postdoctoral Fellowship Award and has received research support from the CIHR, Calorie Control Council, the Canadian Foundation for Dietetic Research (CFDR), and The Coca-Cola Company (investigator initiated, unrestricted grant). He has served as an external resource person to the WHO Nutrition Guidelines Advisory Group (NUGAG), and is the lead author of two systematic reviews and meta-analyses commissioned by the WHO of the relation of saturated fatty acids and trans fatty acids with health outcomes. The WHO paid for his travel and accommodation to attend NUGAG Meetings in Hangzhou, CHINA and Copenhagen, DENMARK. Dr. Kendall has received research support from the Advanced Food Materials Network, Agrifoods and Agriculture Canada (AAFC), Almond Board of California, American Pistachio Growers, Barilla, California Strawberry Commission, Calorie Control Council, CIHR, Canola Council of Canada, The Coca Cola Company (investigator initiated, unrestricted), Hain Celestial, the International Tree Nut Council Nutrition Research and Education Foundation, Kellogg, Kraft, Loblaw Companies Ltd, Orafti, Pulse Canada, Saskatchewan Pulse Growers, Solae and Unilever. He has received travel funding, consultant fees or honoraria from Abbott Laboratories, Almond Board of California, American Peanut Council, American Pistachio Growers, Barilla, Bayer, Canola Council of Canada, The Coca Cola Company, Danone, General Mills, the International Tree Nut Council Nutrition Research and Education Foundation, Kellogg, Loblaw Companies Ltd, Nutrition Foundation of Italy, Oldways Preservation Trust, Nutrition Foundation of Italy (NFI), Orafti, Paramount Farms, Peanut Institute, Pepsi-Co, Pulse Canada, Sabra Dipping Co., Saskatchewan Pulse Growers, Solae, Sun-Maid, Tate & Lyle and Unilever. He is on the Dietary Guidelines Committee for the Diabetes Nutrition Study Group of the European Association for the Study of Diabetes and has served on the scientific advisory board for the Almond Board of California, International Tree Nut Council, Oldways Preservation Trust, Paramount Farms and Pulse Canada. JLS has received research support from the CIHR, Calorie Control Council, The Coca-Cola Company (investigator initiated, unrestricted educational grant), Dr. Pepper Snapple Group (investigator initiated, unrestricted educational grant), Pulse Canada, and The International Tree Nut Council Nutrition Research & Education Foundation. He has received travel funding, speaker fees, and/or honoraria from the American Heart Association (AHA), American College of Physicians (ACP), American Society for Nutrition (ASN), National Institute of Diabetes and Digestive and Kidney Diseases (NIDDK) of the National Institutes of Health (NIH), Canadian Diabetes Association (CDA), Canadian Nutrition Society (CNS), University of South Carolina, University of Alabama at Birmingham, Oldways Preservation Trust, Nutrition Foundation of Italy (NFI), Calorie Control Council, Diabetes and Nutrition Study Group (DNSG) of the European Association for the Study of Diabetes (EASD), International Life Sciences Institute (ILSI) North America, ILSI Brazil, Abbott Laboratories, Pulse Canada, Canadian Sugar Institute, Dr. Pepper Snapple Group, and The Coca-Cola Company. He is on the Clinical Practice Guidelines Expert Committee for Nutrition Therapy of both the CDA and EASD, as well as being on the ASN writing panel for a scientific statement on the metabolic and nutritional effects of fructose, sucrose and high fructose corn syrup. He is a member of the International Carbohydrate Quality Consortium (ICQC) and an unpaid scientific advisor for the ILSI North America, Food, Nutrition, and Safety Program (FNSP). His wife is an employee of Unilever Canada.

## Authors’ contributions

Conception and design: LC, JLS. Analysis and interpretation: LC, JLS. Drafting of the article: LC. Critical revision of the article for important intellectual content: LC, VH, RJdS, CWCK, JLS. Final approval of the article: LC, VH, RJdS, CWCK, JLS. Guarantor, JLS.

## Letter to the editor regarding

Lakhan SE and Kirchgessner A. The emerging role of dietary fructose in obesity and cognitive decline. Nutrition Journal. 2013, 12:114*.*
